# Factors affecting decision making among nurse managers working in government hospitals

**DOI:** 10.3389/frhs.2024.1475402

**Published:** 2025-01-15

**Authors:** Wubete Abeje, Belachew Tegegne, Zenebe Tefera, Yosef Zenebe, Wondwossen Yimam, Birhanu Desu, Yismaw Andargie, Muluken Amare, Molla Kassa, Mulugeta W/Selassie

**Affiliations:** ^1^ Department of Adult Health Nursing, Wollo University, Kelem-Meda, Ethiopia; ^2^ Department of Nursing, Injibara University, Koso-Ber, Ethiopia; ^3^ Department of Midwifery, Wollo University, Kelem-Meda, Ethiopia; ^4^ Department of Psychiatry, Wollo University, Kelem-Meda, Ethiopia; ^5^ Department of Comprehensive Nursing, Wollo University, Kelem-Meda, Ethiopia; ^6^ Department of Emergency and Critical Care Nursing, Wollo University, Kelem-Meda, Ethiopia; ^7^ Department of Pediatrics and Child Health, School of Medicine, Wollo University, Kelem-Meda, Ethiopia; ^8^ Department of Pediatrics and Child Health Nursing, Wollo University, Kelem-Meda, Ethiopia

**Keywords:** decision-making, factors, governmental hospitals, nurse managers, Ethiopia

## Abstract

**Background:**

Effective nursing management and leadership are essential for the provision of desired patient care that will contribute to the improvement of any country's health indicators. However, nurses' views and experiences on the multitude of personal and organizational factors which may impact their decision-making abilities are often neglected in the literature. The study aimed to assess magnitude of poor decision making and its associated factors among nurse managers in South Wollo Zone Governmental Hospitals, Amhara Regional State, Ethiopia, 2023.

**Methods:**

Non experimental cross-sectional study was conducted among 168 nurse managers in South Wollo Zone Public Hospitals from April 01 2023 to May 15/2023. Participants were selected by using a simple random sampling technique. The data were collected by using structured questionnaire from the study participants. Data were entered using EPI data version 4.6 and exported to SPSS version 26 for analysis. The bi-variable logistic regression analysis model was used to identify the potential predictor variable, with *p*-value <0.25 was fitted into the multivariable logistic regression analysis model; *p*-value less than 0.05 and an adjusted odds ratio (AOR) with a 95% confidence interval (CI) was used to declare, factors associated with the outcome variable. The model fitness was checked by using the Hosmer and Lemeshow test. Data were presented with frequency tables, graphs, and pie charts.

**Results:**

The study found that the overall magnitude of poor decision-making among nurse managers was 35.7%. Being self-confident [AOR = 0.01, 95% CI: (00.002, 0.05)], receiving feedback [AOR = 0.24, 95% CI: 0.08, 0.76], and getting managerial support [AOR = 0.22, 95% CI: (0.06, 0.81)] were negatively associated with poor decision-making among nurse managers.

**Conclusion:**

Self-confidence, receiving feedback, and getting managerial support were variables significantly associated with poor decision-making. Authors strongly emphasize providing managerial support for nurse managers, increasing their habit of receiving feedback from colleagues, and encouraging them to build their self-confidence.

## 1 Introduction

### 1.1 Background

Decision-making is an inherent, complex, and vital component of the work of managers (1, 2). Clinical decision-making is an essential component of professional nursing care and, nurses' ability to make effective clinical decisions is the most important factor affecting the quality of care (3, 4). Healthcare decision-makers need to make the best choices for allocating resources, selecting treatments, triaging patients, and choosing affordable interventions. Nurses progress through various management levels, with increasing responsibilities. Staff Nurses provide direct patient care. Head Nurses/Team Leaders supervise small groups and coordinate patient care. Nurse Managers/supervisors oversee unit operations, including staffing and budget. The Matron holds the highest position, leading and overseeing all aspects of the nursing department. Nurse Managers' decisions are influenced by various factors, including organizational culture, resource availability, and workload, communication, and individual characteristics. Nurse managers' decisions are also influenced by the complex interactions and relationships between various public institutions and the people they serve (4). The ability to carry out competent decision-making is a critical and fundamental aspect of professional nursing. Decision-making abilities distinguish professional nurses from ancillary healthcare workers (5). Nurse Managers have an appointed management position within the organization with responsibilities to perform specific tasks (6). Nurse managers employ various decision-making strategies, including intuitive, rational, and clinical decision-making. Intuitive decision-making relies on gut feelings and past experiences for quick judgments, while rational decision-making involves a systematic approach of gathering information, analyzing options, and selecting the most logical choice. Clinical decision-making integrates both intuitive and rational approaches, considering clinical expertise, patient factors, and evidence-based guidelines to make informed decisions. Effective decision-making by nurse managers is essential for improving patient care, job satisfaction, and contributes to the improvement of patient care (6).

### 1.2 Statement of the problem

Nurse managers face numerous challenges in clinical decision-making, including time constraints, complex patient cases, limited resources, uncertainty, ethical dilemmas, communication issues, cultural sensitivity, and personal biases. These factors can impact the quality of care and patient outcomes (7, 8).

A crucial component of nurse management is decision-making, which affects both the general performance of the organization as well as the standard of patient care. Nonetheless, a number of issues that nurse managers frequently deal with may influence how they make decisions. Comprehending these elements is essential to raising the standard of decision-making and, eventually, improving patient outcomes. Numerous characteristics, including as organizational environment, leadership style, workload, and resource availability, have been identified by prior research as potentially influencing the decision-making of nurse managers (9). The precise elements that influence nurse supervisors' decision-making at government hospitals require more investigation, especially in South Wollo Zone.

The nursing discipline's pursuit of professional recognition also relies heavily upon the ability of practicing nurses to correctly define and solve problems which are uniquely nursing in origin (3, 7). In the healthcare system, nurse managers' participation in decision-making is invaluable in preserving cost-effective service and safe patient care. Although nurse managers can ensure optimal healthcare service, researchers have not studied their involvement in decision-making extensively (4). More importantly, understanding which factors to consider when making decisions is crucial for the overall decision-making process. When making a decision, people consciously choose to behave or think in a certain way (2, 4).

Additionally, the active participation of nurse managers in decision-making processes has a profound impact on nurses' attitudes and beliefs, fostering a sense of ownership and commitment to organizational and professional goals. This collaborative approach is indispensable for ensuring the delivery of high-quality healthcare services and facilitating the ongoing professional development of nurses (4, 10). Hence, the study aimed to assess magnitude of poor decision making and its associated factors among nurse managers in South Wollo Zone Governmental Hospitals.

### 1.3 Significance of the study

The primary reason for focusing on government hospitals in the South Wollo zone is their significant role in providing healthcare services to a large population with huge area coverage in the region. These hospitals are representative of the healthcare system in the area and offer a valuable opportunity to study the factors affecting decision-making among nurse managers in a diverse and challenging setting, and moreover, no similar study was conducted in the study area.

So, it is important to investigate the elements that influence nurse managers' decision-making in government hospitals for a number of reasons. First of all, it shows the intricate interactions between administrative, organizational, and personal elements that impact healthcare decision-making. Second, it emphasizes how crucial sound decision-making is to guaranteeing both organizational effectiveness and the best possible patient care. Through the identification of critical elements, this study can offer policymakers, hospital administrators, and nurse managers' important information to better patient outcomes, optimize resource use, and improve decision-making processes. In the end, this research advances nursing management and leadership by encouraging a more patient-centered and evidence-based approach to healthcare delivery.

Hence, the study aimed to assess factors affecting decision-making among nurse managers in South Wollo Zone Governmental Hospitals, Amhara Regional State, Ethiopia, 2023.

## 2 Materials and methods

### 2.1 Study setting

There are 14 government hospitals in the South Wollo Zone. The study was conducted in all 14 South Wollo Zone government hospitals, which are found in Amhara Regional State, Ethiopia. The total number of nurse managers among the 14 government hospitals in South Wollo Zone public hospitals was 256.

### 2.2 Study design

A non-experimental descriptive cross-sectional study was conducted.

### 2.3 Study subjects

All nurse managers who were working in South Wollo Zone government hospitals. All randomly selected nurse managers in South Wollo Zone government hospitals were available during the study period.

### 2.4 Inclusion criteria

All randomly selected nurse managers in South Wollo Zone Government hospitals with 6 months and above work experience in different managerial levels.

### 2.5 Exclusion criteria

Randomly selected nurse managers who were unable to respond due to serious medical or surgical illness during the data collection period and those who are under 6 months of work experience were excluded from the study.

### 2.6 Sample size determination

The sample size was calculated using Taro Yamane's formula (11) to get a reliable sample size from a given population with a 95% confidence level and 5% margin of error.

$$n=\frac{N}{1+N{\left(e\right)}^{2}}$$

where: *n* = sample size

*N* = total population of the study

*e* = the margin of error


$$\frac{256}{1+256(0.05)2}=156.09\approx 156$$

By considering a 10% non-response rate, the sample size would be 156 + (156*10%) = 172.

### 2.7 Sampling techniques and procedures

Convenience sampling technique was used to select study participants. There was a total of 14 government hospitals in the South Wollo Zone. Study samples were selected from each government hospital proportional to their size allocation. The sample size for each hospital was computed by multiplying the total number of nurses for each hospital. Next, the total sample size was determined using a single mean formula, and then divided by the total number of nurses from 14 hospitals. *n* = Nf × nt/Nt. Then participants in each hospital used the proportional allocation formula. *n* = total sample size of each hospital. The study was conducted from April 01 2023 to May 15/2023.

### 2.8 Study variables

#### 2.8.1 Dependent variable

Nurse Managers decision (Good/Poor).

#### 2.8.2 Independent variables

Socio-demographic characteristics: Age, sex, religion, ethnicity, marital status, education level, monthly income, and residence.

Personal factors: Decision maker's physical and emotional state, communication skills, self-confidence, receiving feedback, fear of risk, work experience, prior training, and attending courses in management.

Organizational factors: Place of work, workload, lack of resources, current position, organizational structure, managerial support, and access to information.

#### 2.8.3 Operational definitions

Nurse managers: Nurses in hospital management positions such as matron, head nurse, and supervisors (12).

Decision-making: It is defined as participation in a mental process that results in the selection of a belief or course of action from among various possible alternative options. Decision-making can be assessed by 10 items using a mean score (7, 12).

Good decision-making indicates a higher-than-average response rate of participants to decision-making related questions and, poor decision-making indicates that the participation rate in decision-making is equal or lower than the mean score value (7, 12).

### 2.9 Data collection procedures

Self-administered, semi-structured questionnaires consisting of socio-demographic characteristics, involvement in decision-making, and associated factors were developed from different literatures (4, 13) and used to collect the required data from the study participants. The questionnaire had three components (11 items for socio-demographic characteristics, 10 items for involvement in decision-making, and 15 items for associated factors related to nurse managers' involvement in decision-making) and developed by reviewing the literature of similar studies from previous years (4, 13), Responses for involvement in decision-making were given on a 5-point Likert (extremely poor = 1, poor = 2, neutral = 3, good = 4, and extremely good = 5). The scores of the items were computed by adding the score on each of the 5 styles items, resulting in values from 10 to 50. Finally, those who scored higher than the mean were considered to have good decision-making and participants scored equal to the mean or below the mean were considered as having poor decision-making. The questionnaires were administered by six BSc nurses, and additional two BSc nurse supervisors participated in the data collection process. The study was conducted from April 1, 2023, to May 15, 2023.

In this study, all of the data collectors were trained for 1 week to have a similar concept on the questionnaires that was reviewed by experts (nurse managers) and adopted from previous studies. To ensure the validity of the instrument, it underwent rigorous scrutiny by two esteemed hospital administration experts. In addition, the collected data were checked for completeness and correctness of the information before analysis. Moreover, the questionnaire was pretested on nine (5%) nurses at Kemise Primary Hospital. Fifteen days before the final study. Furthermore, some modifications were made after the pilot test. Cronbach's alpha tests for involvement in decision-making were 0.79 and 0.85, respectively, before and after the test. Besides all these, the data were thoroughly cleaned and carefully entered into Epi-data version 4.6 for the beginning of the analysis in SPSS version-26.

### 2.10 Data processing and analysis

Data were entered into EPI data version 4.6, and then it was exported to SPSS version 26 for cleaning and analysis. Different frequency tables, graphs, and descriptive summaries were used to describe the study variables. Logistic regression was used to identify factors associated with the outcome variable. Bi-variable analysis was carried out to examine the relationship between outcome variables and independent variables by considering *p*-value < 0.25 as a cut-off point for statistical significance. Then multivariable logistic regression was done by considering *p*-value < 0.05 as a cut-off point for statistical significance. The odds ratio was used to measure the strength of the association between dependent and independent variables, and 95% CI was also used to determine the significance of associations.

## 3 Results

### 3.1 Socio-demographic characteristics of the study participants

From a total of 172 participants, 168 nurse managers in South Wollo Zone Government hospitals participated in this study with a response rate of 97.67%. More than half of the study participants were older than 30 years. The majority of the study participants, 146 (86.9%), were male. On the other hand, 76 (45.2%) of the study participants were Orthodox by religion. More than half of the study participants, 100 (59.5%), were married, and the majority of them, 135 (80.4%), were Amhara in ethnicity. The majority of the study participants (94.6%) were urban residents. Nearly fifty-eight percent of the study participants, 99 (58.9%), were diploma holders (Table 1).

**Table 1:** Socio-demographic characteristics of study participants in South Wollo Zone Governmental Hospitals, Amhara Regional State, Ethiopia (*n* = 168).

Characteristics	Category	Frequency	Percent
Age (in years)	21–25 years	21	12.5
	26–30 years	50	29.5
	>30 years	97	58
Sex	Male	146	86.9
	Female	22	13.1
Religion	Orthodox	76	45.2
	Muslim	65	38.7
	Protestant	18	10.7
	Catholic	9	5.4
Ethnicity	Amhara	135	80.4
	Oromo	23	13.6
	Tigray	10	6
Marital status	Single	36	21.5
	Married	100	59.5
	Divorced	21	12.5
	Widowed	11	6.5
Educational status	Diploma	99	58.9
	Degree	58	34.5
	Master	11	6.6
Monthly income	>3,500	168	100
Residence	Urban	159	94.6
	Rural	9	5.4

### 3.2 Decision-making among nurse managers in South Wollo Zone government hospitals

**Figure 1:**
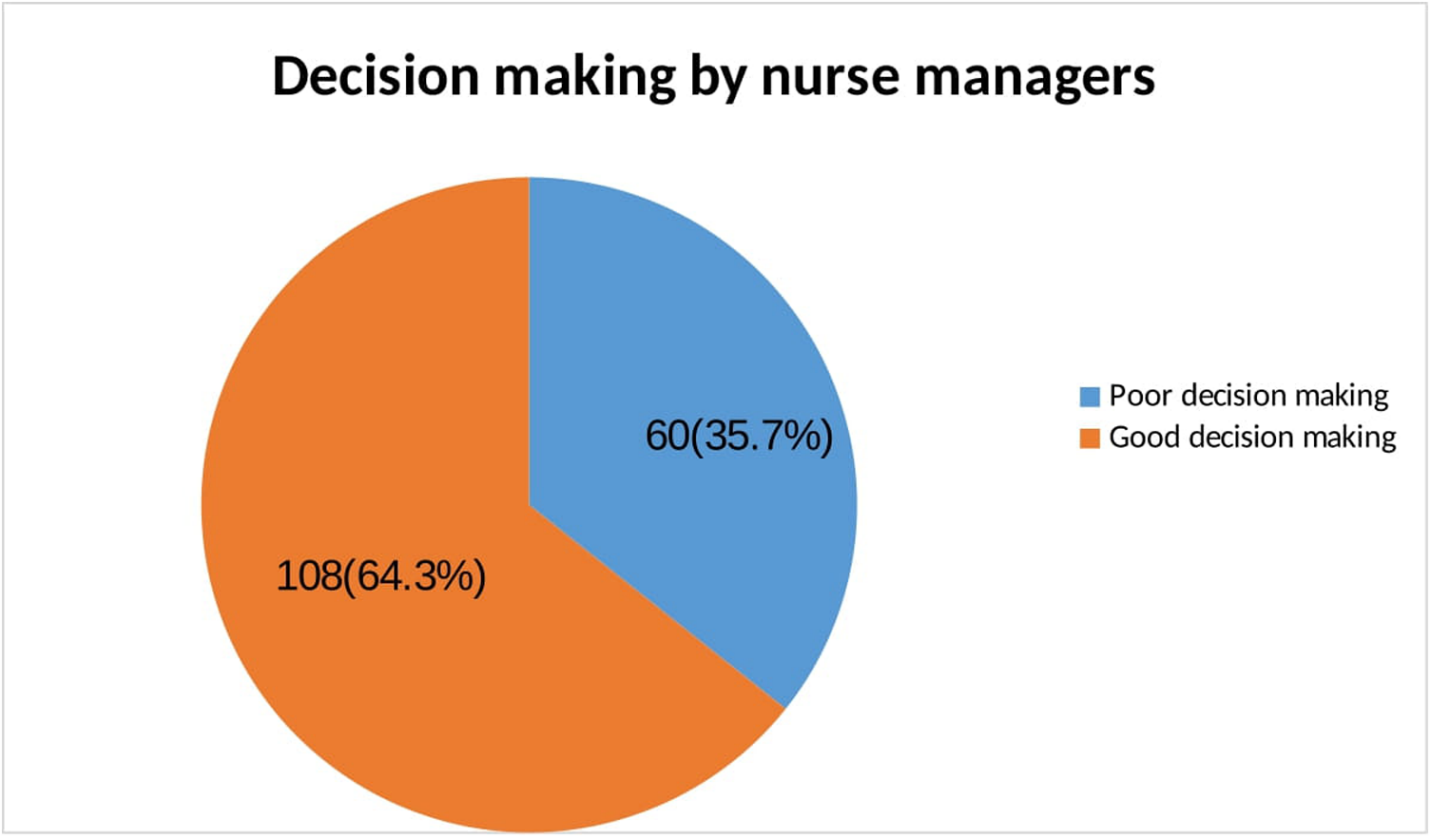
Magnitude of decision making among nurse managers in South Wollo Zone, Amhara Regional State, Ethiopia, 2023.

In this study finding, 60 (35.7%) with a 95% CI [28.1, 43.5] of the study participants had poor decision-making. However, nearly two-thirds of nurse managers reported good decision-making (Figure 1).

### 3.3 Personal factors of the study participants

The majority of the study participants 102 (60.7%) had self-confidence. On the other hand, 109 (64.9%) of the study participants had good communication skills. Of all study participants, 115 (68.5%) of the study participants had received feedback from their colleagues after decision-making was done (Table 2).

**Table 2:** Personal factors affecting decision making among study participants in South Wollo Zone Governmental Hospitals, Amhara Regional State, Ethiopia (*n* = 168).

Characteristics	Category	Frequency	Percent
Having self-confidence	Yes	102	60.7
	No	66	39.3
Communication skills among nurse managers	Yes	109	64.9
	No	59	35.1
Receive feedback from your colleagues	Yes	115	68.5
	No	53	31.5
Fear any risk regarding decision-making	Yes	146	86.9
	No	22	13.1
Attend courses in management	Yes	66	39.3
	No	102	60.7

### 3.4 Organizational factors among the study participants

Almost two-thirds (65.5%) had a workload in their hospital. Of the study participants, 101 (60.1%) had previous experience in nursing management. More than half of the study participants 102 (60.7%) involve other nurses in the decision-making process. On the other hand, most of the study participants, 123 (73.2%) hadn't gotten managerial support from their seniors. Among all study participants, 104 (61.9%) of the study respondents participated in managerial decision-making. Nearly fifty percent of the study respondents had access to information for their decision-making (Table 3).

**Table 3:** Organizational factors that affect the decision-making of nurse managers in South Wollo Zone Governmental Hospitals, Ethiopia (*n* = 168).

Characteristics	Category	Frequency	Percent
Workload in your hospital	Yes	110	65.5
	No	58	34.5
Previous experience in nursing management	Yes	101	60.1
	No	67	39.9
Involve other nurses in the decision-making process	Yes	102	60.7
	No	66	39.3
Have power in decisions related to nursing activities	Yes	70	41.7
	No	98	58.3
Accessibility of resources	Yes	45	26.8
	No	123	73.2
Getting managerial support from top-level managers	Yes	45	26.8
	No	123	73.2
Participate in managerial decision-making	Yes	104	61.9
	No	64	38.1
Access to information	Yes	97	57.7
	No	71	42.3

### 3.5 Current position of nurse managers

**Figure 2:**
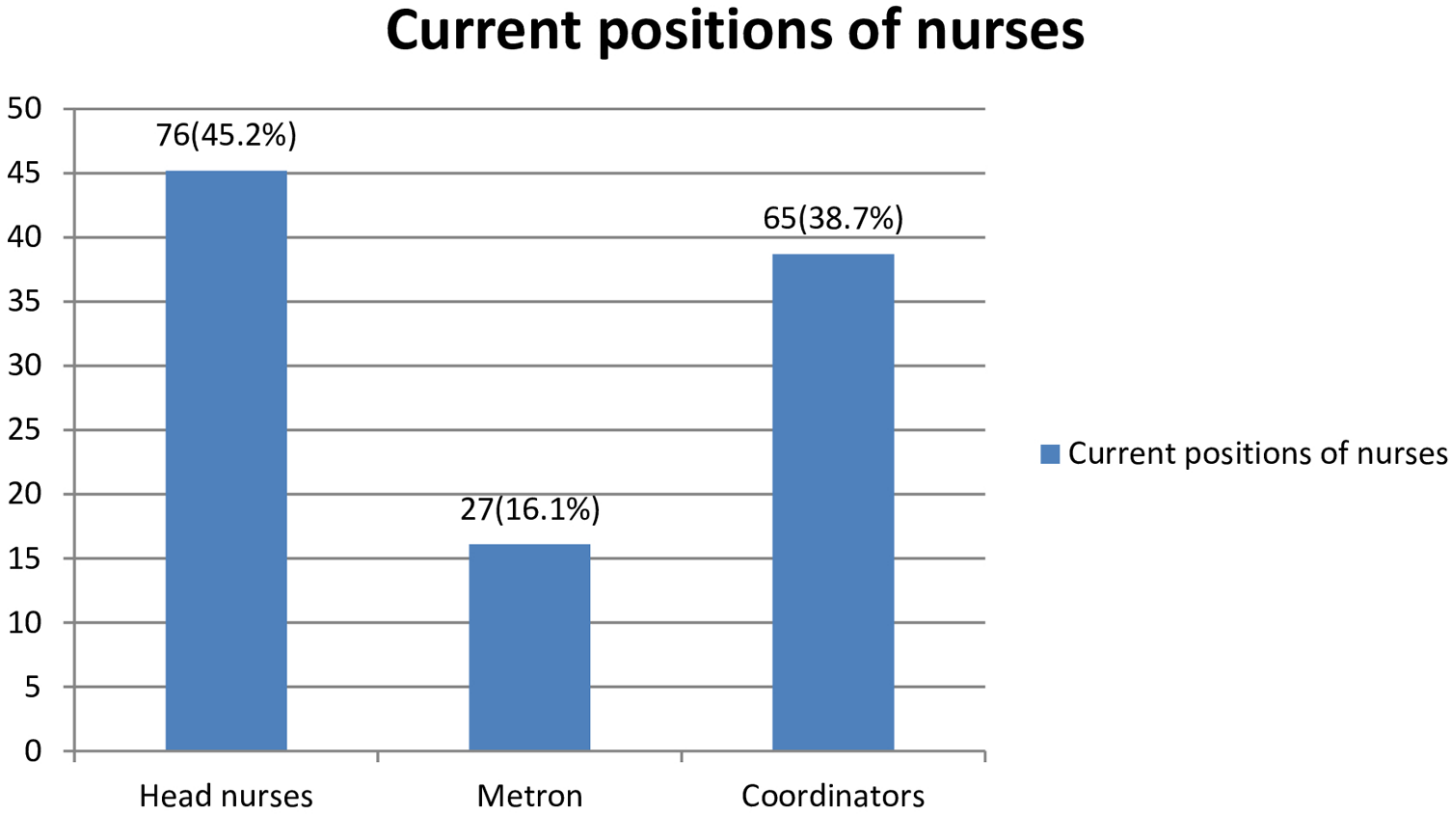
Current nurse manager's position in South Wollo Zone Governmental Hospitals, Amhara, Ethiopia, 2023 (*n* = 168).

From all nurse managers, many of the study participants, 76 (45.2%), were head nurses, followed by coordinators, which are 65 (38.5%) (Figure 2).

### 3.6 Factors associated with decision-making among nurse managers

In bi-variable analysis, seven variables, namely, self-confidence, communication skills, receiving feedback from colleagues, work experience, managerial support, access to information, and age of the study participant, were candidate variables for multi-variable logistic regression at a *p*-value of less than 0.25. In multivariable logistic regression, three of seven variables were significantly associated with decision-making among study participants at a 5% level of significance. The significant factors of decision-making were lack of self-confidence, inability to receive feedback from their colleagues, and managerial support had statistically significant associations with decision-making among study participants. For participants who had no self-confidence, the odds of decision-making were reduced by 99% as compared to those who had self-confidence [AOR = 0.01, 95% CI: 0.002, 0.05]. For those who didn't receive feedback from their colleagues, the odds of decision-making were reduced by 76% as compared to those who received feedback from their colleagues [AOR = 0.24, 95% CI: 0.08, 0.76]. On the other hand, for those who didn't get managerial support from top-level managers, the odds of decision-making were reduced by 78% as compared to those who got managerial support from top-level managers [AOR = 0.22, 95% CI: 0.06, 0.81] (Table 4).

**Table 4:** Factors associated with decision-making among nurse managers in South Wollo Governmental Hospitals, Amhara Regional State, Ethiopia (*n* = 168).

Variables	Decision making		COR (95% CI)	AOR (95% CI)
	Poor	Good		
Self-confidence				
Yes	6	96	0.01 [0.005, 0.04]	0.01 [0.002, 0.05]***
No	54	12	1	1
Communication skill				
Yes	21	88	0.12 [0.06, 0.25]	1.12 [0.27, 4.66]
No	39	20	1	1
Work experience				
Yes	24	65	0.44 [0.52, 1.89]	1.50 [0.47, 4.83]
No	36	43	1	1
Receive feedback				
Yes	23	92	0.11 [0.05, 0.23]	0.24 [0.08, 0.76]*
No	37	16	1	1
Get managerial support				
Yes	11	74	0.10 [0.23, 1.06]	0.22 [0.06, 0.81]*
No	49	34	1	1
Get access to information				
No	30	67	0.61 [0.32, 1.16]	1.91 [0.55, 6.63]
Yes	30	41	1	1
Age				
21–25 years	4	17	0.41 [0.75, 7.69]	3.29 [0.47, 3.14]
26–30 years	21	29	1.28 [0.39, 1.57]	1.40 [0.41, 4.8]
>30 years	35	62	1	1

COR, crude odds ratio; AOR, adjusted odds ratio. Hosmer and Leme show goodness of fit test = 0.13.

* *p* < 0.05.

** *p* ≤ 0.001.

*** *p* < 0.0001.

## 4 Discussion

In this study finding, the overall magnitude of poor decision-making among study participants was 35.7% with a 95% CI [28.1, 43.5]. The present finding of this study is lower as compared to the study conducted in Egypt (57.1%) (7). this finding is also lower as compared to the study finding done at Addis Ababa University (57.7%) (8). Another study in Egypt revealed that factors such as inadequate resources, lack of knowledge and skills, and limited support contribute to poor decision-making among nurse managers (14). These discrepancies might be due to differences in organizational structures, hospital policies, individual personality traits, cultural differences, individual problem-solving skills and critical thinking, level of stress, work load, a supportive environment, level of collaboration and communication, technological advancements, and access to resources, data collection methods, having relevant training, selection criteria of participants, and level of education.

In this study finding, self-confidence [AOR = 0.01, 95% CI: 0.002, 0.05] was found to be negatively associated with poor decision-making among nurse managers. Those who hadn't self-confidence had poor decision-making as compared to those who had self-confidence. This finding is in line with the study done in Australia and Jimma University (6, 15). The possible justification might be related to the fact that confident people are more likely to bounce back and that they do so faster after a setback. Their belief in their ability to succeed allows them to do this. They chalk up failures as learning opportunities and use the lessons to build back bigger and better.

Receiving feedback from colleagues [AOR = 0.24, 95% CI: 0.08, 0.76] was negatively associated with decision-making. Those who didn't receive feedback from their colleagues about previously made decisions had poor decision-making as compared to those who had received feedback. This finding is similar to the study done in Addis Ababa (4). This could be because feedback control is a process that managers can use to evaluate how effectively their teams meet the stated goals at the end of a production process. Feedback control evaluates the team's progress by comparing the output the team was planning on producing to what was produced.

The other important variable that showed a statistically significant association with decision-making was the lack of managerial support from top-level managers [AOR = 0.22, 95% CI: (0.06, 0.81]. It is negatively associated with decision-making. Those who hadn't gotten managerial support from top-level managers had poor decision-making as compared to those who had gotten managerial support from their seniors. This study finding is in line with the studies conducted in Addis Ababa and the University of Gondar (4, 16). Those who hadn't gotten managerial support from their seniors had poor decision-making as compared to those who got managerial support from their top-level managers. The study highlights the global need for improved managerial support, training, and feedback for nurse managers to enhance decision-making, the quality of care and improve patient outcomes.

### 4.1 Strengths and limitations of the study

This study employed a multicenter approach with primary data, offers a broader representation of study participants, thereby enhancing the generalizability of its findings. However, the cross-sectional design precludes the establishment of causal relationships. These potential risk factors and possible mechanisms need to be further explored using qualitative studies.

## 5 Conclusion

The overall magnitude of poor decision-making among nurse managers (matron, head nurse, and nurse supervisors) was 35.7%. In this study, self-confidence, receiving feedback from their colleagues, and getting managerial support from their top-level managers were variables that showed a significant association with poor decision-making among nurse managers in South Wollo Zone government hospitals.

## 6 Recommendations

Senior and top-level nurse managers should provide managerial support for nurse managers to maximise their decision-making skills. They should also provide training on managerial functions to develop self-confidence among nurse managers.

Moreover, nurse managers shall be aware of the advantages of receiving feedback from their colleagues on previously made decisions. Healthcare organisations can also enhance the decision-making skills of nurse managers, leading to improved patient care, increased efficiency, and overall organisational effectiveness.

## Data Availability

The original contributions presented in the study are included in the article/Supplementary Material, further inquiries can be directed to the corresponding author.
